# Laparoscopic redo anastomosis for management of intraperitoneal anastomotic leakage after colonic surgery

**DOI:** 10.1186/s12893-022-01555-6

**Published:** 2022-03-25

**Authors:** Yi-Chang Chen, Tao-Wei Ke, Yuan-Yao Tsai, Abe Fingerhut, William Tzu-Liang Chen

**Affiliations:** 1 Department of Colorectal Surgery, China Medical University Hospital, Taichung, Taiwan; 2 Department of General Surgery, Ruijin Hospital, Shanghai Minimally Invasive Surgery Center, Shanghai Jiao Tong University School of Medicine, Shanghai, 200025 People’s Republic of China; 3 China Medical University Hsinchu Hospital, Zubei, Taiwan; 4 Department of Surgery, Medical University of Graz, Graz, Austria

**Keywords:** Laparoscopic, Redo anastomosis, Intraperitoneal leakage, Colonic surgery

## Abstract

**Background:**

There is still no consensus on the management of intraperitoneal anastomotic leakage after colonic surgery. Among of various treatment strategies, laparoscopic redo anastomosis for intraperitoneal leakage has rarely been reported in the literature and is condemned by some. The aim of this study is to demonstrate the feasibility and safety of laparoscopic redo anastomosis for intraperitoneal anastomotic leakage.

**Methods:**

Retrospective chart review of laparoscopic redo anastomosis for intraperitoneal anastomotic leakage after colonic surgery from January 2013 to May 2020. An accompanying video demonstrates the technique.

**Results:**

Fifteen consecutive patients underwent laparoscopic redo anastomosis for management of leakage after colonic surgery; two patients required conversion to open repair. A protective stoma was created in three patients during the second operation. There was no re-leakage nor mortality in this series.

**Conclusions:**

Laparoscopic redo anastomosis was feasible and safe for the management of intraperitoneal anastomotic leakage after colonic surgery. Considering the advantages of re-do laparoscopy, this procedure should be part of every surgeon’s armamentarium to deal with anastomotic leakage and represents a logical alternative to the “Diversion and Drainage” technique.

**Supplementary Information:**

The online version contains supplementary material available at 10.1186/s12893-022-01555-6.

## Introduction

Anastomotic leakage occurs in approximately 1–19% of patients after colorectal surgery [1], less commonly for intraperitoneal (1–4%) than for extraperitoneal anastomoses (5–19%) [1]. Among the causes of anastomotic leakage, mechanical failure (insufficient suture purchase, inadequate suturing technique or knotting, or faulty staple formation) usually leads to early (within seven days post-operative) anastomotic break-down [2].

While laparoscopy has been shown to be feasible for the management of colorectal anastomotic leakage, most published studies, however, deal with the “Diversion and Drainage” technique [3, 4]. Laparoscopic redo anastomosis is rarely reported in the literature [3–6], compared to open surgery, and is condemned by some [7]. The aim of this study is to demonstrate the feasibility of laparoscopic redo anastomosis and report the outcome in 15 consecutive patients.

## Materials and methods

Between January 2013 and May 2020, of 2449 patients who had laparoscopic colonic surgery with anastomosis in our unit, 35 patients underwent laparoscopic re-exploration due to anastomotic leakage: 15 had laparoscopic redo anastomosis and form the study population. Demographic data and perioperative outcomes were reviewed retrospectively. An intraperitoneal leak was defined as any anastomotic site that communicated with the peritoneal cavity above the peritoneal pouch. Leakage was diagnosed via drainage, imaging, or laparoscopic reintervention. The purported mechanism of failure was assessed for all patients by scrutinous review of the original anastomosis thanks to routine video recording of all operations in our unit. Regular discussion among our surgical team as to the possible cause of mechanical failure (tension, insufficient suture purchase, inadequate suturing technique or knotting, or faulty staple formation) was a prerequisite to this indication. Redo anastomosis was the first choice for early management of intraperitoneal leaks in patients who had stable clinical status and viable bowel, bowel wall edema permitting, mechanical (staple or suture) failure confirmed by intra-operative findings and a critical review of the original operative video. However, “Diversion and Drainage” was indicated when the anastomosis defect was minor (< 1 cm) with inoperable phlegmon while the Hartmann procedure was reserved for patients with defects > 1 cm or when they were unstable.

### Surgical technique

Laparoscopic re-exploration is the standard approach for patients undergoing laparoscopic colorectal surgery who have a confirmed or suspected anastomotic leak in our unit. The technical steps of laparoscopic redo anastomosis were recorded (Additional file 1: video S1). Stoma formation was dependent on surgeon preference and/or patient clinical status. Abdominal drainage was left routinely after redo anastomosis.

## Results

From January 2013 to May 2020, after revision of the original operation and agreement among the surgeons that the anastomotic leakage was due to mechanical failure rather than ischemia (n = 5) or other patient-related factors (n = 15), 15 consecutive patients underwent laparoscopic redo anastomosis for management of leakage after colonic surgery. Patient characteristics and operative details are described in Table 1. End-to-end anastomosis was performed for patients undergoing anterior resection while side-to-side anastomosis was performed in the other procedures. All initial anastomoses methods were mechanical. The median Mannheim Peritonitis index was 20 (range 14–38). The median delay before the re-do operation was 5 (2–15) days.

**Table 1:** Patient demographics and initial operation details

Patient	Age	Sex	ASA	Type of surgery	Interval between first and second operations (days)	Mannheim peritonitis index	Leakage site
1	57	Female	3	Right hemicolectomy	3	26	Anterior
2	64	Female	2	Left hemicolectomy	4	20	Posterior
3	64	Male	2	Right hemicolectomy	5	21	Angle
4	38	Male	2	Transverse colectomy	5	20	Total dehiscence
5	55	Male	2	Left hemicolectomy	2	27	Anterior
6	59	Male	2	Left hemicolectomy	7	25	Posterior
7	65	Female	2	Left hemicolectomy	7	18	Posterior
8	57	Male	2	Right hemicolectomy	7	15	Anterior
9	75	Female	2	Anterior resection	5	14	Total dehiscence
10	57	Female	2	Transverse colectomy	4	20	Anterior
11	83	Female	2	Left hemicolectomy	4	26	Anterior
12	71	Female	3	Right hemicolectomy	5	21	Angle
13	35	Male	2	Left hemicolectomy	15	16	Posterior
14	80	Male	3	Anterior resection	6	38	Total dehiscence
15	65	Female	2	Right hemicolectomy	7	20	Posterior

Eleven patients underwent segmental bowel resection including the anastomosis while four patients required extended right hemicolectomy (Table 2). Three patients had a protective stoma during redo surgery. Two patients required conversion to laparotomy due to bowel distension and/or dense adhesions. The median operation time was 270 (100–560) minutes (Table 2). After the second operation, there were no re-leakage nor re-operations required. One patient needed ventilator support in ICU for 3 days. There was no post-operative mortality and median hospital stay was 12 (8–40) days (Table 2).

**Table 2:** Second operation details and outcome

Patient	Surgery type	Anastomosis	Protective stoma	Conversion	Operation Time (minutes)	Postoperative complication	Hospital stay
1	Segmental resection	Ileocolic	No	No	240	Ileus	10
2	Segmental resection	Colocolic	No	No	180	No	9
3	Segmental resection	Ileocolic	No	Yes	250	Ileus	20
4	Extended RH	Ileocolic	No	No	340	No	8
5	Segmental resection	Colocolic	No	No	180	No	13
6	Extended RH	Ileocolic	No	No	360	No	12
7	Segmental resection	Colocolic	No	No	420	No	16
8	Segmental resection	Ileocolic	No	No	270	No	9
9	Segmental resection	Colocolic	No	No	100	No	8
10	Extended RH	Ileocolic	No	No	330	Ileus	16
11	Extended RH	Ileocolic	Yes	No	540	Gastric ulcer	21
12	Segmental resection	Ileocolic	No	No	260	No	14
13	Segmental resection	Colocolic	Yes	Yes	560	No	12
14	Segmental resection	Colorectal	Yes	No	220	Pneumonia, Renal failure, heart failure	40
15	Segmental resection	Ileocolic	No	No	450	No	12

*RH* right hemicolectomy

## Discussion

Laparoscopic redo anastomosis is feasible and safe in selected patients (early reoperation and mechanical anastomotic failure without ischemia) with intraperitoneal anastomotic leakage after colectomy. None of the 15 patients had re-leakage, 12 without a protective stoma; stoma closure was performed 3 months after laparoscopic reintervention for the others. No death occurred.

There is still no consensus on how to best manage anastomotic leakage after colonic surgery; strategies depend on patient clinical status, bowel viability, surgeon preference, experience and technique, as much as current beliefs [7].

Traditionally both patient status and the existence of peritonitis have dictated whether it is safe to preserve the anastomosis, perform a resection and end-colostomy or drain [7]. Several authors have reported a higher mortality when the MPI was 21 or over [8]. Of note in our study, seven of the 15 patients had a MPI of 21 or greater, but no death occurred. Of note, the dogma of no anastomosis in peritonitis was laid down many years ago, when diversion in times of war-fare was a time-, labor- and personnel-sparing solution for penetrating colonic injuries [9]. Trauma surgeons were among the first to contest this policy and advocate that resection and anastomosis was feasible and safe [10]. More recently surgeons have argued that resection and anastomosis could be performed safely in patients with Hinchey III or even IV diverticulitis [11, 12]. Therefore, our results challenge traditional thinking and the decision of whether to resect and divert or redo the anastomosis should take into account the bowel status and the mechanism of anastomotic failure, in addition to patient status. In our series, the decision to redo the anastomosis was taken only after consensus among our team that the leakage was due to mechanical failure (revisualization of the initial operation and careful inspection the anastomotic site) and the reintervention took place early (within 1 week) after the initial operation.

Among of various treatment strategies for leakage, redo anastomosis is the most challenging and time consuming. Redo anastomosis has most often been reported via laparotomy [7] but large series are sparse. Rickert et al. reported 67 patients who underwent open reintervention; however, only three patients had redo anastomosis [7]. However, laparoscopic redo anastomosis for management of leakage after colonic surgery is rarely reported in the literature [3–6]. The main advantage of laparoscopic exploration for anastomotic leakage is to avoid relaparotomy, a procedure that increases the rate of wound complications and duration of hospital stay [3–5]. In two series, no re-anastomosis was performed [3, 4]. Lee et al. reported 15 laparoscopic re-operations after colonic surgery (of 61 in all); however, whether the leaks were intraperitoneal, whether the authors repaired the leaks and the exact number of laparoscopic redo anastomosis are difficult to determine from the data provided. Another advantage of re-do laparoscopy is the possibility of re-performing the anastomosis intracorporeally. Compared to re-do anastomosis via laparotomy, laparoscopy reduces the need for colonic mobilization, avoiding excessive traction on the intestines and vessels to exteriorize the segments and entails a smaller mini-laparotomy wound [13].

Laparoscopy is routinely used for re-exploration in our unit. We believe that early diagnosis and reintervention were key to our high success rate. Our median interval between initial surgery and reintervention (5 days) was shorter than that [8.6 (4–113) days] reported by Rickert et al. [7]. While “early” re-intervention was defined as six days or less from initial surgery [2], four patients in our series successfully underwent laparoscopic re-anastomosis on post-operative day 7. This underscores that a specific timeline is perhaps too strict; colonic viability and type of anastomotic failure (mechanical or ischemic) are more important factors to consider. On the other hand, delayed reintervention could result in dense adhesions and therefore the need to convert to laparotomy. This is illustrated by our 13th patient, referred from other hospital 15 days after initial surgery, who had to be converted to open due to dense adhesions. Of note, however, even this patient had a successful recovery after re-anastomosis.

After redo anastomosis for management of leakage, a protective stoma is routinely used after colorectal anastomotic leakage [3–7]. Rickert et al. concluded that anastomosis repair or redo anastomosis without stoma formation was a cause of failure [7]. Conversely, in the series of Lee et al., three of six in open anastomotic repair and one of twelve in laparoscopic repair had an uneventful recovery, but the reasons for this choice were not mentioned [3]. In our study, a protective stoma was not routinely constructed after redo colonic anastomosis. The reasons were that surgical re-intervention took place early; the bowel was not swollen and was clearly viable. Secondly, judged by routine careful scrutiny of video recordings of the initial operation, a protective stoma could be omitted if technical failure (and not patient factors) was the main reason for initial colonic anastomotic leakage.

Some may argue that redo anastomosis without stoma goes against the standard surgical treatment such as Hartmann’s procedure or “Diversion and Drainage” technique in peritonitis. However, of note, we were unable to find any objective or factual data that determine when to perform a stoma after colonic resection and anastomosis, only personal recommendations.

This was a single center, non-comparative, relatively small series. Larger series are necessary to confirm our results. We did not assess colonic vascularization during the operation other than visually. We recognize that surgeons are poor in judging colonic vascularization visually [14] and in the future, intra-operative assessment by ICG might be an interesting avenue to explore.

## Conclusion

Laparoscopic redo anastomosis was feasible and safe for the management of intraperitoneal leakage after colonic surgery when performed early. The technique is illustrated in our video and provided promising outcomes in our series. Protective stoma formation was not routinely performed after colonic anastomotic leakage repair and therefore may be reserved for selected patients or surgeon preference.

## Supplementary Information

**Additional file 1.** Laparoscopic redo anastomosis in three cases.

## Data Availability

Data sharing is not applicable to this article as no datasets were generated or analyzed during the current study.
